# Is noninvasive hemoglobin measurement suitable for children undergoing preoperative anesthesia consultation?

**DOI:** 10.1007/s10877-024-01194-7

**Published:** 2024-07-20

**Authors:** Katja Mohnke, Julia Smetiprach, Yuri Paumen, Philipp Mildenberger, Yannick Komorek, Eva-Verena Griemert, Eva Wittenmeier

**Affiliations:** 1 Department of Anesthesiology, University Medical Center of the Johannes Gutenberg University Mainz, Mainz, Germany; 2 Institute of Medical Biostatistics, Epidemiology and Informatics, University Medical Center of the Johannes Gutenberg University Mainz, Mainz, Germany

**Keywords:** Anemia, Hemoglobin, Children, Accuracy, Noninvasive hemoglobin measurement, Patient blood management

## Abstract

Preoperative anemia in children is a significant clinical concern requiring precise diagnosis. Although traditional blood sample collection is effective, it poses challenges because of children’s aversion and technical difficulties. Therefore, this study explores the suitability of noninvasive hemoglobin measurements in children during preoperative anesthesia consultation. Noninvasive hemoglobin measurement (SpHb®; Masimo) in children aged ≤ 17 years was performed during preoperative anesthesia consultation and compared with laboratory hemoglobin (labHb) levels. SpHb was measured in 62 children (median age: 6 years, standard deviation [SD] ± 5.3) without adverse effects but was unsuccessful in one child. The bias, limits of agreement, and root mean square error between SpHb and labHb were 0.3, −2.26– +2.8, and 1.3 g/dl, respectively. LabHb demonstrated a significant regression relationship with R^2^ of 0.359. LabHb was associated with a negative effect on bias [− 0.443 (CI 95: − 0.591– − 0.153, P < 0.001)], i.e., SpHb tends to underestimate labHb for high labHb values. The retest reliability between two consecutive SpHb measurements was 0.727 (P < 0.001). Double measurement of SpHb, age, weight, sex, heart rate, and perfusion index had no significant effects on accuracy. Using SpHb, a specificity of 96.3% (95% confidence interval [CI 95]: 87.3%–99.5%) and a sensitivity of 57.1% (CI 95: 18.4%–90.1%) were observed. Based on adapted cut-off values for SpHb (age-dependent cut-off values plus 0.8 g/dl), a sensitivity of 100% (CI 95: 64.6%–100%) was achieved for the investigated study collective. SpHb measurement in children is a quick procedure. The accuracy of hemoglobin measurement is insufficient for the diagnosis of anemia. Thus, whether the calculated cut-off SpHb values of this study collective can be considered for anemia screening in pediatric patients undergoing preoperative anesthesia consultation should be confirmed. *Trial registration number and date of registration*: This prospective study was registered at ClinicalTrials.gov (NCT03586141).

## Introduction

Preoperative anemia is associated with poor clinical outcomes of noncardiac surgery in neonates [1] and children [2, 3]. Anemia, which is characterized by reduced age-dependent hemoglobin (Hb) levels [4], is a significant problem in preschool children with a prevalence of 42.6% worldwide and 22.9% in Europe [5]. Hence, patients with anemia who are scheduled for major surgery should be identified as early and precisely as possible for diagnosis and treatment. This is reflected as a central point of patient blood management, an interdisciplinary concept aimed at the rational use of blood products in the perioperative context to reduce transfusion-associated increased morbidity and mortality in children [6–8].

Generally, anemia is diagnosed based on low Hb levels after blood sampling. This invasive procedure is often uncomfortable for children [9] and can be challenging to perform. Frequent blood draws also increase the risk of iatrogenic anemia [10].

Thus, for detecting anemia in pediatric patients without the adverse effects of invasive blood sampling, noninvasive Hb measurement (SpHb) is a promising method to increase patient safety and is particularly recommended by the Society for the Advancement of Patient Blood Management for the detection of anemia in children [6].

The Masimo Pronto® Pulse CO-Oximeter® is a noninvasive spectrophotometry-based handheld device. Compared with continuous SpHb measurements, it is a spot-check device that measures hemoglobin at a certain time. The manufacturer indicated that this device improved Hb detection performance from 6 to 11 g/dl [11]. In adults, Masimo Pronto® showed high levels of usability, acceptability [12], and patient comfort [13]. To date, most studies have described the Masimo Pronto® device as not sufficiently accurate [12–14]. However, by defining new cut-off values, the device can be used as a screening test for adults to index anemia with high sensitivity [15, 16].

To date, a few studies have evaluated the agreement between Masimo Pronto® and laboratory hemoglobin (labHb) values in children [12, 17, 18], and, to the best of our knowledge, no study has investigated the feasibility of noninvasive hemoglobin measurement in children as a diagnostic tool for preoperative anemia. Therefore, data are necessary to assess accuracy and, if feasible, to define a new SpHb cut-off value that can be evaluated in prospective studies.

Therefore, this study aimed to evaluate the feasibility, and accuracy of preoperative hemoglobin measurement in pediatric patients undergoing elective surgery using Masimo Pronto®.

## Methods

This prospective study was conducted from July to October 2018 after obtaining approval from the local ethics committee (Rhineland-Palatinate, Germany; 2018-13157-Epidemiologie). The data of the adult patient collective, which was collected using a comparable methodology, was previously reported [15].

Written informed consent was obtained from all legal guardians of the participating patients and the patients aged ≥ 14 years. The study was conducted during preoperative anesthesia consultation at the Department of Anesthesiology of the University Medical Center of the Johannes Gutenberg University Mainz, Germany. All patients who underwent preoperative anesthesia consultation before elective surgery were eligible.

Hemoglobin measurements were performed noninvasively using the SpHb measurement device Masimo Pronto®, which employs Masimo SET® Measure-through Motion and Low Perfusion™ Pulse oximetry and rainbow® technology for spot-check measurements of total hemoglobin (Masimo Corporation, Irvine, California, USA). The reusable DCI Mini Sensor was used as the sensor.

Two measurements were performed successively, and the sensor was replaced in the second measurement.

The sensor clip was placed based on a priority list following the manufacturer’s recommendations and depending on the child’s adherence and signal quality. For children weighing > 10 kg, it was placed on the little finger of the nondominant hand, and if measurement was not possible, it was measured on either another finger of the nondominant hand, a finger of the dominant hand, or a toe. For children weighing from 3 to 10 kg, it was measured on the big toe, the thumb, or any other finger. For each measurement, the time required to read the SpHb value after the sensor was recorded.

LabHb was measured by evaluating a venous blood samples using a hematology analyzer (Siemens Advia®2120, Munich, Germany). Blood samples were collected in the surgical ward, within 24 h of the noninvasive measurement. When no blood sample was obtained within this timeframe, noninvasive hemoglobin measurement was repeated. If no blood sample was obtained, the patient’s data were removed from further data analysis.

To diagnose anemia, age-dependent lower hemoglobin limit values indicated by the World Health Organiziation (WHO) were used (Table 1). The primary endpoint of this prospective investigation is the agreement between labHb and SpHb values.

**Table 1:** Age-dependent cut-off values for anemia diagnosis according to the World Health Organization (WHO) [4]

Population	Hemoglobin cut-off value for anemia [g/dl]
Children aged 6–59 months	11.0
Children aged 5–11 years	11.5
Children aged 12–14 years	12.0
Girls aged ≥ 15 years	12.0
Boys aged ≥ 15 years	13.0

The secondary end-points were the time it took to measure SpHb, the reliability of SpHb, the influence of patient and measurement factors on the accuracy of SpHb, the sensitivity and specificity of SpHb predicting anemia and the definition of a new cut-off SpHb value to detect true anemia with higher sensitivity.

### Statistical analysis

Statistical evaluation was performed using IBM SPSS Statistics (IBM SPSS Statistics for Windows, version 20. IBM Corporation, Armonk, NY, USA). For the pediatric study participants [15], a sample size of n = 60 was determined necessary to obtain a 95% confidence interval (CI 95) for the difference between two measurements with a width of at most 60% of the SD with a probability of 95%.

Bland–Altman analysis was used to examine the accuracy of SpHb. The first of the two noninvasive measurements was used for all SpHb calculations, except for the analysis of reproducibility and area under the curves (AUC) values. It visualizes bias, defined as the mean difference between the reference and the alternative methods, and limits of agreements (LOAs), defined as mean bias ± 1.96 times the SD of the bias [19, 20], the range where 95% differences between the measurement methods are expected to occur. Agreement between SpHb and labHb levels was further assessed using the root mean square error (RMSE), percentage of outliers, and the receiver operating characteristic (ROC) curve with CIs. The graphical error grid analysis is based on Morey, Gravenstein [21], based on a manuscript by Clarke, Cox [22]. Here, the collected values are displayed in an error grid, showing the absolute values of the noninvasive haemoglobin measurement and those of the reference method, as well as the difference between these values. The clinical significance of the difference, as assessed by experienced clinicians, is highlighted in color. Due to the age-dependent cut-off values for anemia diagnostics, this has been adapted accordingly in this manuscript.

The manufacturer of the noninvasive method states an accuracy of ± 1 g/dl [11]. Consequently, the percentage of outliers was defined as the proportion of measurements that deviated below 1 g/dl, 1–2 g/dl, 2–3 g/dl and 3–4 g/dl compared to the measurements of the reference method. The retest reliability of SpHb and the correlation between bias and defined influencing variables were tested using Pearson’s correlation coefficient [23]. The AUC values were reported with 95% CIs using the DeLong approach [24]. Improvements in accuracy were assessed using the average of both SpHb measurements instead of the first measurement using the DeLong test for ROC correlation. Simple linear regression analyses were conducted to examine the impact of age, weight, sex, SpO_2_, perfusion index, and heart rate on the difference between labHb and SpHb levels.

AUC values were calculated for a modified predictor variable, i.e., the difference in SpHb measurement from the lower cut-off value to diagnose anemia. This was conducted to incorporate the age-dependent definition of anemia as reported by the WHO [4]. All ROC analyses were conducted using R [25], with the R package pROC [26].

P < 0.05 was accepted as indicative of statistical significance.

## Results

SpHb levels were measured in 62 children and compared with that of labHb levels. Among the children, 11% were anemic. Noninvasive measurement was unsuccessful in one child because of device malfunction. This child’s data were excluded from further analysis.

No adverse events associated with SpHb or labHb measurements occurred. It took 16.7 s (SD ± 2.44) before the signal of the first SpHb measurement could be recorded. For five children (8.1%), the measurement location of the sensor was changed for a valid first measurement. All patient and measurement characteristics are presented in Table 2.

**Table 2:** Patient and measurement characteristics

Parameter	Quantity
Sex	Female 21 (34%)
	Male 41 (66%)
Age [years]	6* (0–17)
Height [cm]	116.5* (72–182)
Weight [kg]	19.5* (8–80)
ASA physical status	
1	20 (32%)
2	33 (53%)
3	9 (15%)
Anemic patients	7 (11%)
LabHb [g/dl]	12.7* (7.7–16)
SpHb [g/dl]	13.0* (8.7–16.5)
Duration of measurement [s]	16.8* (14–30)
Number of attempts until the first measurement signal	1.081* (1–2)
Perfusion index	5.7* (0.7–16.0)
Heart rate [bpm]	98.4* (55–138)
SpO_2_ [%]	97.9* (89–100)

Values are presented as absolute numbers or medians (marked with an asterisk). The corresponding percentage share or minimum to maximum is shown in parentheses

*ASA* American Society of Anesthesiologist, *LabHb* laboratory hemoglobin measured using a hematology analysis, *SpHb* noninvasive hemoglobin, *SpO*_*2*_ oxygen saturation

**Fig. 1:**
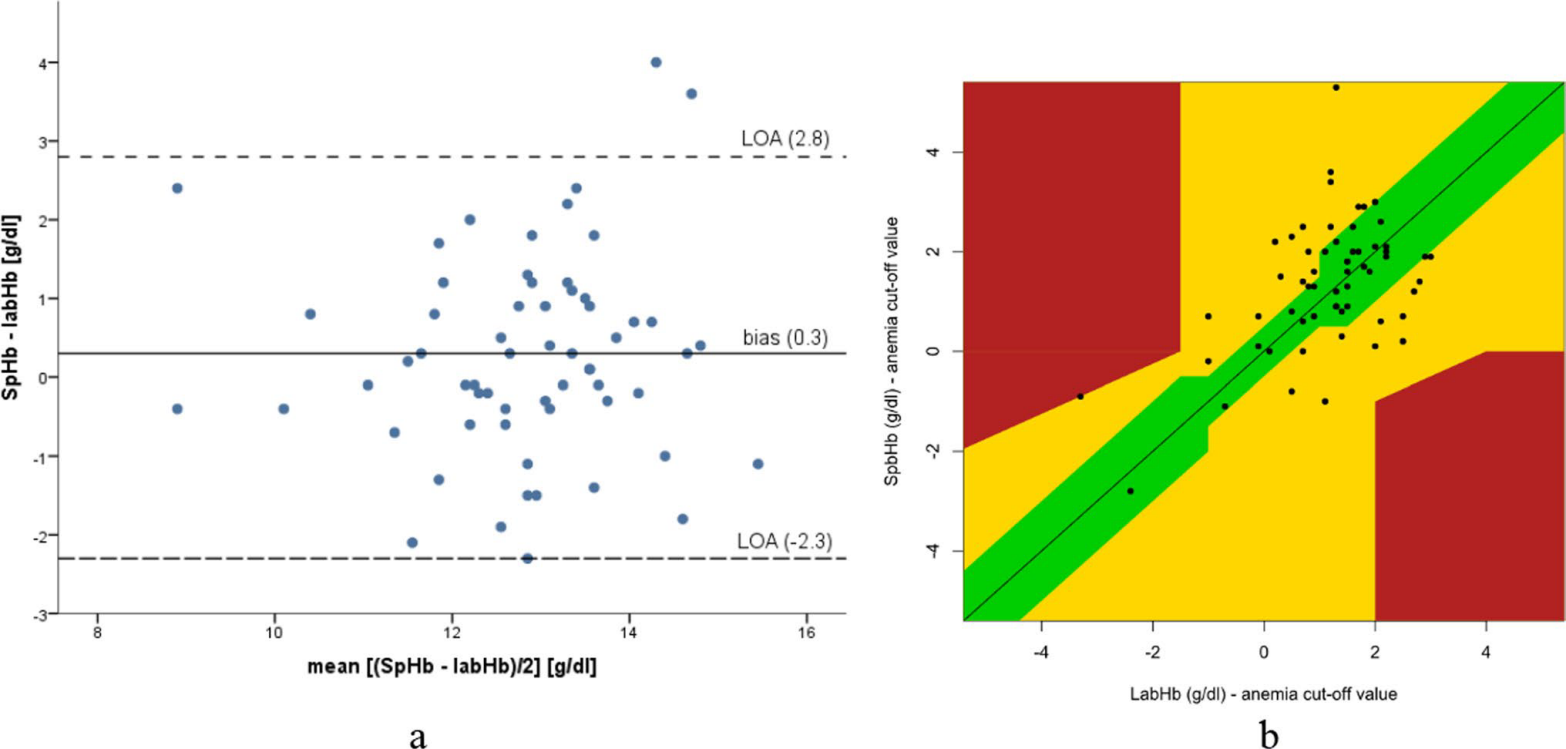
**a** Bland–Altman diagram showing the agreement between SpHb and labHb. **b** Error Zone Approach according to Morey et al. [21], adapted by us for children. The reference method (labHb) is shown on the abscissa and the alternative method (SpHb) on the ordinate; both minus the age-dependent cut-off value for the diagnosis of anemia according to WHO [27]. The color coding in green, yellow and red corresponds to the authors’ assessment of clinical significance. If the measured value is in the green zone, there is probably no relevant clinical misinterpretation of the hemoglobin value. The yellow zone creates significant errors in hemoglobin measurement, although the magnitude of these errors is not as great as those in the red zone. In the red zone, major therapeutic errors can occur, posing substantial risks to patients. *labHb* laboratory hemoglobin measured using a hematology analyzer, *SpHb* noninvasive hemoglobin, *LOA* limits of agreement

The agreement between SpHb and labHb using a Bland–Altman plot is presented in Fig. 1a; the corresponding error zone approach graphic is shown in Fig. 1b. Bias, LOA, RMSE, and percentage of outliers are shown in Table 3.

**Table 3:** Comparison of SpHb and labHb

	Comparison SpHb/labHb
Bias	+ 0.3 g/dl (CI 95: − 0.07– + 0.59)
Limits of agreement	− 2.26 (CI 95: − 2.83– − 1.69) to + 2.8 (CI 95: + 2.21– + 3.34) g/dl (CI 95: − 2.26– + 2.78)
Root Mean Square Error	1.3 g/dl
Out of range (|SpHb-LabHb|≤ 1 g/dl)	37 (60.7%)
Out of range (|SpHb-LabHb|> 1 and ≤ 2 g/dl)	17 (27.7%)
Out of range (|SpHb-LabHb|> 2 and ≤ 3 g/dl)	5 (8.2%)
Out of range (|SpHb–LabHb|> 3 and ≤ 4 g/dl)	2 (3.3%)

*labHb*, laboratory hemoglobin measured using a hematology analyzer; *SpHb*, noninvasive hemoglobin. *CI 95*, 95% confidence interval

Pearson’s correlation coefficient between the first and second SpHb measurements was 0.727 (CI 95: 0.589–0.988, P < 0.001).

**Fig. 2:**
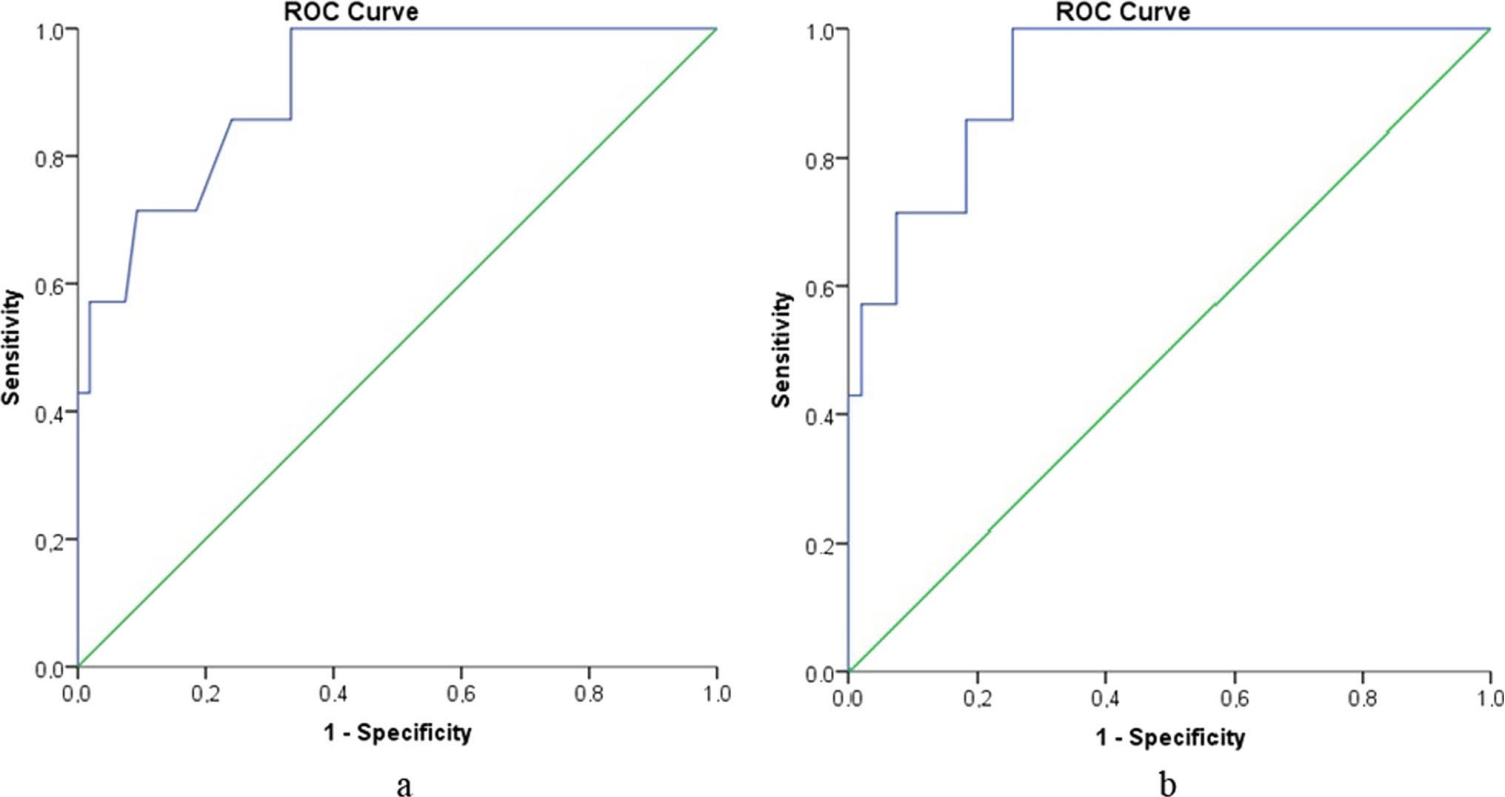
Comparison of the accuracy of one **a** versus the mean of two consecutive **b** SpHb measurements. For the first measurement, the area under the ROC curve (AUC) was 0.907 [CI 95: 0.802–1.0], for the mean of two consecutive measurements, the area under the AUC was 0.925 [CI 95: 0.842–1.0]. Although the mean of the two measurements produced a higher AUC than the first measurement alone, the DeLong test did not show a significant improvement; the difference in AUCs was 0.013 [CI 95: − 0.016; 0.04, P = 0.38]. *SpHb* noninvasive hemoglobin

The mean of two consecutive SpHb measurements resulted in a lower deviation of the mean and a smaller fluctuation range: the bias and LOAs between SpHb and labHb were 0.12 g/dl (CI 95: − 0.14– + 0.38) and − 1.9 (CI 95: − 2.31– − 1.42) to 2.1 g/dl (CI 95: 1.66–2.55), respectively. We examined whether the mean of two consecutive SpHb measurements provided a significantly better agreement with labHb than a single measurement (Fig. 2).

Table 4 presents the agreement between SpHb and labHb values in a four-field table.

**Table 4:** Agreement between SpHb and labHb

	True normal	True low
SpHb normal	52	3
SpHb low	3	4
	Specificity 96.3% (CI 95: 87.3%–99.5%)	Sensitivity 57.1% (CI 95: 18.4%–90.1%)

*LabHb* laboratory hemoglobin using a hematology analyzer, *SpHb* noninvasive hemoglobin, *CI 95* 95% confidence interval

labHb below the age-dependent cut-off values by the WHO is considered true low. Values above this are considered true normal. Values are absolute numbers. Specificity and sensitivity to predict anemia are given in %

The linear regression analysis revealed that age, weight, sex, heart rate, and perfusion index had no significant effects on the bias.

LabHb exhibited a significant regression relationship with R^2^ of 0.359. LabHb was associated with a negative effect on bias (− 0.443 [CI 95: − 0.591– − 0.153, P < 0.001]), i.e., SpHb tends to underestimate labHb for high labHb values.

With a prevalence of 11.29% for anemia in the study collective, using SpHb, the specificity was 96.3% (CI 95: 87.3%–99.5%) and the sensitivity was 57.1% (CI 95: 18.4%–90.1%) (Table 4).

Calculations were performed to determine a lower SpHb cut-off value that could be used to detect anemia with high sensitivity. For the initial SpHb measurement, the AUC was 0.927 [0.852–1]. The Youden index was maximized at a cut-off value of 0.8 g/dl (i.e., if the SpHb value was lower than the cut-off value for age-dependent anemia diagnosis in [27] + 0.8 g/dl), resulting in a sensitivity of 100% [CI 95: 64.6%–100%] and a specificity of 79.6% [CI 95: 67.1%–88.2%].

## Discussion

This study including 62 children showed that using the Masimo Pronto® device for noninvasive hemoglobin measurement during preoperative anesthesia consultation was feasible; however, the agreement with labHb and the sensitivity to detect anemia were poor. By defining a lower cut-off value for SpHb as the age-dependent hemoglobin value for anemia plus 0.8 g/dl, a sensitivity of up to 100% (CI 95: 64.6%–100%) could be achieved, making it conceivable as a screening tool.

The feasibility of the Pronto® device was satisfactory in children. With an average of 16.7 s, a rapidly available measurement signal was achieved, and sensor replacement was rarely necessary. The failure rate of measuring SpHb in our patients was low. Thus, in elective cases involving older children, a measurement signal could be obtained significantly more frequently than that in the pediatric intensive care unit (PICU) patient population [18]. This could be because PICU patients with critical illness have limited perfusion, in contrast to the collective examined here. Moreover, without adverse events, the application proved to be safe.

The bias between SpHb and labHb was + 0.3 g/dl. However, the LOA (− 2.3– + 2.8 g/dl) shows a wide range. This range makes the device unsuitable for detecting anemia when used as a measurement device. This wide range of LOA is comparable to previous pediatric studies using Masimo Pronto® in various settings, in which its lack of accuracy has been debated [12, 16, 18]. This trend is also evident in the adult collective of measurements performed with this device in the preoperative anesthesia consultation [15]: In women, bias and LOA were 0.3 g/dl (− 2.3– +2.8 g/dl) and in men 0.1 (− 2.6– +2.8 g/dl). In their meta-analysis, Hiscock et al. reported comparably wide LOAs (− 3– +2.9 g/dl) with newer Masimo devices, primarily in an adult patient population [28], whereas the manufacturer states an accuracy of ± 1 g/dl [29]. However, in our study, 39.2% of SpHb values deviated by > 1 g/dl from labHb. We consider that these deviations are not sufficiently accurate for anesthesia consultation.

Although the retest reliability was good, repeated measurements did not improve accuracy. Because the time available during the anesthesia consultation and the patience of children are limited, double measurement of the SpHb value cannot be recommended.

Age did not influence accuracy. Hsu et al. [17] demonstrated slightly better accuracy in a younger study group but did not consider age as a specific factor influencing measurement accuracy. The perfusion index did not affect bias in this study. To the best of our knowledge, no other study has highlighted the influence of the perfusion index on bias in infant SpHb measurements. A higher bias is expected to be associated with a poorer measurement signal. In general, the perfusion index was sufficiently high in this study. The device provider recommends a value of 1 [29].

LabHb had a significant impact on SpHb. SpHb tends to underestimate labHb for high labHb values. This observation has already been reported in previous studies involving adult patient populations [15].

The sensitivity of SpHb was poor at 57.1% (CI 95: 18.4%–90.1%), making it inadequate for diagnosing anemia solely based on SpHb measurement. However, the specificity was relatively high at 96.3% (CI 95: 87.3–99.5). In clinical practice, this approach may allow for the preselection of nonanemic children, thereby reducing the need for further invasive diagnostic procedures.

In this study, a lower SpHb cut-off value was calculated after data collection. This lower cut-off value can possibly serve as a screening test, indexing “anemia” with high sensitivity, when SpHb values remain under the calculated SpHb cut-off value. At an age-dependent cut-off value of hemoglobin + 0.8 g/dl, the sensitivity could reach 100% but with a wide range in the CI 95 of 64.6%–100%. Hsu et al. [17] examined 110 children during a well-child examination using Masimo Pronto® for a lower cut-off value. With a lower bias of − 0.066 g/dl and a narrower LOA of − 2.124– +1.992, they set the age-independent lower cut-off for SpHb to 11.5 g/dl, achieving a sensitivity of 82% (CI 95: 75%–90%) and a specificity of 65% (CI 95: 56%–74%). The average age of the examined children was 14.5 months, which was significantly younger than the patient population examined in this study. Currently, data to prospectively evaluate a cut-off value in the pediatric patient population are lacking during preoperative anesthesia consultation. Prospective studies should be conducted to validate these calculated cut-off values for detecting pediatric anemia with the Error Grid Approach presented here potentially being incorporated into clinical decision-making.

This study has some limitations. The prevalence of anemia in the study population was 11%, which is lower than that in Europe (22.9%) [5]. This lower prevalence may have contributed to a wider spread of the data. The timing of the SpHb and labHb measurement was not standardized within a time frame of 24 h. In young adults, it is known that the hemoglobin content in the blood varies over the course of the day [30]. We contemplate that this assumption can also be transferred to our study group, for example in the context of changes in the intravascular volume and hydration stage during the course of the day. The fact that SpHb and labHb were not measured simultaneously but up to a 24-h interval, may also have caused a difference in labHb and SpHb values. This is a consequence of the study design, yet it also mirrors clinical reality in preoperative anesthesiology consultations.

To the best of our knowledge, this is the first study to investigate the suitability of noninvasive hemoglobin measurement in children during preoperative anesthesia consultation.

## Conclusion

The accuracy of noninvasive hemoglobin measurement using Masimo Pronto® was not sufficient for diagnosing pediatric anemia in the present study; accordingly, it is not suitable for use in children during preoperative anesthesia consultation. Therefore, clinicians are advised not to rely solely on the device for preoperative anemia diagnosis. However, it is simple and quick to use and should be considered as a screening tool in future studies with suitable, age-adapted cut-off values in the preoperative pediatric patient population, provided that device-related improvements result in better accuracy.

## Data Availability

Data can be provided upon reasonable request by the corresponding author. No datasets were generated or analysed during the current study.
